# The Use of “Tail-Pedometers” to Evaluate the Impact of Dipterans in Feeder Cattle

**DOI:** 10.3390/insects13070616

**Published:** 2022-07-08

**Authors:** Marc Desquesnes, Kornkanok Thaisungnoen, Piangjai Chalermwong, Adèle Nevot, Clément Fossaert, Antoine Lucas, Sathaporn Onju, Phoompong Boonsaen, Sathaporn Jittapalapong

**Affiliations:** 1 CIRAD, UMR InterTryp, ENVT, 23 Chemin des Capelles, 31300 Toulouse, France; 2 InterTryp, Univ Montpellier, CIRAD, IRD, F-34398 Montpellier, France; adele.nevot@inserm.fr (A.N.); clement.fossaert@gmail.com (C.F.); antoine.lucas@agrocampus-ouest.fr (A.L.); 3 Faculty of Veterinary Medicine, Kasetsart University, Bangkok 10900, Thailand; kornkanok.tha@gmail.com (K.T.); joy_piangjai@hotmail.com (P.C.); 4 Department of Entomology, Faculty of Agriculture at Kamphaeng Saen, Kasetsart University, Kamphaeng Saen 73140, Thailand; camba-11@hotmail.com; 5 Department of Animal Sciences, Faculty of Agriculture at Kamphaeng Saen, Kasetsart University, Kamphaeng Saen 73140, Thailand; fagrppb@ku.ac.th; 6 Faculty of Veterinary Technologies, Kasetsart University, Bangkok 10900, Thailand; fvetspj@ku.ac.th

**Keywords:** *Stomoxys*, tabanids, mosquitoes, defense movements, economic loss, tail flicks

## Abstract

**Simple Summary:**

Hematophagous flies are a pest for livestock due to their bites, annoyance and the diseases they transmit. Cattle exposed to biting flies exhibit defence movements such as tail flicks. The evaluation of biting fly density and annoyance by counting tail flicks of cattle has been validated in the past, but it is highly time consuming. In this study, we evaluated the use of sport pedometers attached to the tails of feeder cattle, in order to evaluate tails flick frequency in two groups of six feeder cattle: Group A was protected by a mosquito net, and Group B was in open-air. Tail flicks were also recorded visually. In addition, insect density was evaluated using three fly traps in the surrounding of Group B. A strong correlation was observed between fly density and visual records of tail flicks; a stronger correlation was found between fly density and tail pedometer records. The reports of tail flicks at night were even able to draw attention to a huge infestation by mosquitoes. Finally, in these experimental conditions, tail pedometers proved to be useful tools in evaluating density and the impact of dipterans on cattle behaviour. They will be useful tools to evaluate new biting fly control methods.

**Abstract:**

Hematophagous flies are a pest for livestock; their direct impact reduces productivity, and they are vectors of parasites, bacteria and viruses. Their control using insecticides is inefficient and highly polluting. The validation of new control tools requires efficacy and cost-effectiveness evaluation. The quantification of hematophagous insects’ impact in livestock is a challenging prerequisite. Tail flicks counts can reliably evaluate fly-burden; however, visual records are tedious and time-consuming. In the present study, automation of tail flick counts was made through the use of pedometers attached to the tail, in two groups of feeder cattle. Group A was kept in a pen under the protection of a mosquito net, and Group B was kept in an open-air pen. The fly density of Group B was evaluated using fly traps. The apparent density per trap ranged from 130 to 1700 in the study. The mean pedometer records per 24 h ranged from 957+/−58 bits in Group A to 11,138+/−705 bits in Group B. The night/day records observed in Group A (200/800 bits) were drastically increased in Group B (1000–4000/4000–14,000 bits) and variable along seasons. A very high correlation was observed between fly density and visual records or pedometer records (PR). Two-hour PRs proved to be a reliable predictive tool for fly density. Moreover, the pedometers revealed an unsuspected but significant nuisance of mosquitoes, which should be thoroughly investigated.

## 1. Introduction

A large range of hematophagous insects of the order Diptera are pests of livestock [1]. From the tiniest midges to the largest tabanids, from facultative to obligatory hematophagous, and from diurnal to nocturnal, their relative impact on livestock production is suspected to be high, but not clearly and fully determined [1]. It is generally accepted that the direct impacts of hematophagous Diptera (annoyance, pain, stress, blood despoliation, etc.) are linked with hematophagy, biting characteristics, feeding behavior, size and abundancy of the insects. The most important species of hematophagous Diptera annoying livestock belong to the sub-orders Brachycera and Nematocera. In the sub-order Brachycera, tsetse flies are low prolific insects, restricted to Africa. Other Brachycera flies are more prolific and cosmopolite: tabanids, stomoxyine flies, hippoboscids and the common flies of the genus *Musca*, which also includes some strictly hematophagous species such as *Musca crassirostris* [1,2]. The sub-order Nematocera contains the very common and abundant mosquitoes (Culicidae) and the biting midges: culicoides, phlebotomes and simulie flies [1]. Overall, it is generally accepted that the dipteran pests exerting the most important direct impact are the diurnal hematophagous Brachycera flies, which are large in size [1]. Although tabanids, stomoxyine flies and mosquitoes are found practically everywhere on earth, the presence and abundance of the others vary from a place to another. Not only acting as direct pests, these insects are also responsible for the biological or mechanical transmission of a large range of pathogens, such as bacteria (*Coxiella burnetii*/Q fever, *Bacillus anthracis*/Anthrax, *Anaplasma*, etc.), viruses (responsible for blue tongue, equine infectious anemia, Japanese encephalitis, etc.) and parasites (*Trypanosoma* spp., *Besnoitia*, etc.) [3,4].

Even if control methods do exist, they are inefficient, convenient or safe. Protecting livestock from Diptera using mosquito nets is a simple option; however, it is generally difficult to maintain in the farms, due to the movements of animals and the management of food, manure and others. Smoke may be used to repel the insects, especially during the peak of crepuscular activity; however, it is not fully efficient and can only delay the contact between insects and livestock [5], although their efficacy was demonstrated, especially for tsetse flies [6,7]. Killing the insects coming into contact with livestock by the use of insecticides in pour-on, bath, spray or injection form is another option. The main drawbacks of these methods are their high cost and very short length of action [8,9], which increase the frequency and, thus, the (i) environmental toxicity, (ii) animal product contamination and (iii) development of resistance to insecticides [10,11,12]. Other means of control such as traps and/or impregnated screens have been used [13] and a multi-target method based on insecticide-impregnated screens (*FlyScreens*) was recently developed for the control of these pests [14].

The main dipteran pests of livestock, *Musca* spp., *Stomoxys* spp., tabanids and mosquitoes, are highly diverse in genera and species, and they are present all over the world [1]. Their population dynamics are highly variable, but they generally exhibit a seasonal peak of high density, most often for 3–5 months, when they constitute a temporary high burden for livestock [15,16]. 

In order to protect themselves from hematophagous insects, animals exhibit defense movements such as hear and head shaking, tail flicks, skin shaking and leg-kicks [17]. This behavior leads to a loss of energy and tends to reduce the time used for feeding, potentially leading to a decrease in food intake [18]. Under controlled conditions, stable flies released in a screened enclosure reduced the dry matter intake of cattle and also impacted the dynamic of ingestion (faster rates of intake and shorter feeding times were recorded) [19]. Such parameters might then be measured when evaluating the impact of flies on livestock.

The defense movements of cattle exposed to the attack of flies can easily be observed in the fields; amongst them, tail flicks are the most obvious and frequently seen. Three decades ago, in an attempt to develop the awareness of flies’ annoyance and to measure their impact on cattle in French Guiana, the methodology of tail flick counts was established by visual records [18,20]. A mathematical relation was drawn between the number of tail flicks observed per minute and the apparent density of tabanids, evaluated using fly traps. The behavior of cattle was modified by high fly density, inducing increased tail flick frequency, gathering of animals for mutual protection, and modification of the timeline of food activity [20]. Other authors, in Southern California, observed that, under a moderate fly pressure, dairy cattle tail flicks were not effective to repel biting flies (*Stomoxys*), but were the easiest way to quantify flies annoyance and help monitoring pest activity [17]. Tail flick counts proved to be a reliable data to estimate biting-insects density and thus the potential impact of flies on cattle. However, counting the defense movement of cattle by visual observation is a heavy task, highly time-consuming, difficult to implement on free or semi-free animals, due to their displacements and the fact that they can hide each other. In addition, individual data brought by visual records are limited in time, because they require the observation of a single cattle by a single technician. For these reasons, few studies were carried out using this method.

In a study submitted (https://assets.researchsquare.com/files/rs-717630/v1/5ea15e73-0352-47b4-be29-a9fd4b8f8a6d.pdf?c=1631886882; accessed on 28 June 2022), we have evaluated the impact of dipteran insects on Kamphaeng Sean bull-calves growing performances by comparison of feed intake, feed performances and weight gains of two groups of feeder cattle, exposed or not to the insects. The economic impact was drawn out and related to the fly density measured around the cattle pens using Vavoua and Nzi traps. In the present paper, we develop and validate a new methodological tool to measure a defense movement of cattle against biting flies. For automation of tail flicks counts, we used small “sport-pedometers” attached to the tail of cattle in two groups of feeder cattle placed either in fly-proof conditions or in open-air, under the attacks of the natural dipteran population. 

## 2. Material and Methods

### 2.1. Study Area, Equipment and Food Delivering

This study was carried out in the Kamphaeng Sean Campus of Kasetsart University, Ban Yang district, Nakhon Pathom Province (Thailand) (latitude 14.02022; longitude: 99.964187), between August 2016 and March 2017, on two groups of young feeder cattle 9–11 months old at the beginning of the experiment.

The two groups of six animals were separated in two neighboring pens (A and B) of 84 sqm each, delimited by metallic barriers, equipped with food and water troughs and weighting devices. Pen B was a normal open-air area. Pen A was made fly-proof by the use of a mosquito net set-up at one meter around, with a 2.5–3 m high ceiling and an airlock entrance. If flies were found inside the mosquito net, they were cached and killed every day to make sure this pen was permanently fly-less. The animals received pellet food with hay and water ad libitum. 

### 2.2. Animal Selection and Grouping 

Twelve animals, 9–11 months old, were selected out of a group of 30 Kamphaeng Sean bull-calves [21], from the Animal sciences breeding unit (Faculty of Agriculture, Kamphaeng Saen Campus, Kasetsart University). First, all calves were kept free in an open-air 300 sqm park and observed daily for 3 days by two technicians, to score the attractivity and the behavior of the animals towards the flies. Attractivity was estimated by the mean of 3 repeated counts of the total flies visible on one side of each animal, morning and evening, for 3 days; animals exhibiting extreme values were rejected. Reactivity/passivity was estimated by observation of defense movements; increasing intensity was scored from 0 to 4 at the same time as the insects counts. Animals exhibiting extreme reactivity (mean score >3) or extreme passivity (mean score <1) were rejected from the experiment. Animals showing aggressive behavior amongst each other or towards the manipulators were also rejected. Non-rejected animals were weighted (using the TAN Scale Model LP7110C-1T, Locosc Ningbo Precision Technology Co., Ltd., Jiangsu, China) before the selection was completed. Age and weight were then considered, and animals with extreme values were excluded. Remaining candidate animals were submitted to blood tests for hemoparasites by blood smear observation, hematocrit centrifuge technique [22], Enzyme Linked Immuno-Sorbent Assay (ELISA) against *Trypanosoma evansi* infection, Card Agglutination Test for Trypanosomes/*T. evansi* (CATT *T. evansi*) methods [23,24] and PCR for Trypanozoon infection, using TBR primers in protocols previously published [25,26]. Only animals resulting negative to all tests were kept for the experiment. Twelve bull-calves were selected as described above and randomly split into two groups of 6 animals (Group A and B), considering the age and weight of the animals, to ensure the two groups exhibited similar mean weight and mean age. These animals were treated against intestinal and blood parasites with Aben-15^®^ (150 mg Albendazole; F.E. Pharma Company Limited^®^, Bangkok, Thailand) and Ivomec^®^ Plus (1% Ivermectin and 10% Clorsulon, Merial^®^ Inc., Duluth, GA, USA). The animals were introduced into a pen (A or B) at the end of week 2 of each experiment (the first two weeks being during the preliminary period, when all animals were kept in open-air).

### 2.3. Trapping Method and Insect Counts

Because the most important Dipteran pests of livestock are the large-size hematophagous Brachycera flies, Vavoua and Nzi traps, which are fairly adapted to such flies, were used [27]. Once a week, in the pasture next to the pens, two Vavoua traps and one Nzi trap were set up on a line, at 60 m apart and the trap’s cages were installed and changed, for 6 trapping sessions of 2 h, from 06:00 to 18:00. Cages were then collected at 8:00, 10:00, 12:00, 14:00, 16:00 and 18:00, to evaluate temporal distribution of fly density along the days [28,29]. Insects were identified using keys for *Musca*, tabanids and Stomoxyine flies [30,31,32]. For this study, only genus identification was made at the exception of *Muscra crassirostris*, identified to the species, in order to distinguish from non-obligatory hematophagous *Musca* spp., recorded as “common flies”. Insect records (IR) were made as follows: hematophagous flies (tabanids + stomoxyine flies + *M. crassirostris*), common flies (*Musca* spp others than *M. crassirostris*) and total flies. IR were defined as the total number of insects trapped by the 3 traps in a unit of time. IR2 (2 h Insect Record) was defined as the total number of insects caught in the three traps during a 2 h trapping session; such records were implemented during the 6 daily sessions previously described. IR12 was defined as the total number of insects caught by the three traps over 12 h in a day (from 6:00 to 18:00). Total number of insects trapped during each of the 6 trapping periods were compared with the tail flick counts recorded during the same periods of time, in order to demonstrate the correlation between fly density and tail flick counts (see below for tail flick counting methods). In some instances, we present insect density in a more usual manner, as the “apparent density per trap” (ADT), which is usually calculated as the total insect record (IR) divided by the total number of traps [33]: three in the present case. In our study, because we were using 2 types of traps (2 Vavoua and 1 Nzi), we used either IR or “ADT”.

In the midterm of the experiment, one night-time mosquito trapping session (from 18:00 to 06:00) was organized in order to evaluate the potential role of mosquitoes in the annoyance of cattle, using a CDC trap [34] hung 2.5 m high in the center of pen B. The trapping box was changed every two hours for 6 trapping periods. Culicidae and other night flies possibly annoying cattle were identified and counted [35,36]. The results were compared with data obtained from the tail pedometers during the same 6 time periods. 

### 2.4. Tail Flick Counting Methods 

Because the methodology of tail flick counts was established by visual records [18,20], we used the visual records as a reference method. 

Visual records (VR): The cattle tail flick counts were estimated by sessions of visual records using a manual counter for 5 min, by one technician, for each of the 12 animals. These visual records (VR5m) were implemented 6 times a day, once during each of the 6 trapping periods of 2 h. Each animal was then observed for a total of 30 min/day (6 × 5 min), in a protocol derived from previously published results [20]. The exact times at the beginning of each VR5m (accuracy of a minute, for example, 16:35) were registered to further compare VR5m scores with PR5m, the data obtained from the electronic devices (pedometer records = PR) for the same period of 5 min (in our example, 16:35–16:40).

Pedometer records (PR): Fitbit zip^®^ pedometers (manufactured by FitBit^®^ Inc., 199 Fremont Street, San Francisco, CA 94105, USA) are developed for human sports and activity monitoring; they are waterproof, powered by disposable batteries and they continuously record movements (“steps” or “bits”). Step counts can be downloaded, after Bluetooth synchronization using an electronic device (smartphone or computer), from the FitBit^®^ website. Fifty Fitbit zip^®^ pedometers were purchased for this study.

One pedometer was attached to the tail of each animal, at a distance of 15–20 cm from the basis of the tail, using a regular gaze-band first to protect the skin, placing the pedometer and covering it with an elastic gaze-band, and further fixed on by a sticky gaze-band (Neotape 2^®^). Once a week, the sticky-bands were renewed and the battery of pedometers checked and replaced when necessary. The bit-counts (so-called “steps” recorded by the pedometers) of the animals were downloaded and saved three times per week, using the Fitbit^®^ smartphone application, for further data analysis. Continuous data obtained from the electronic devices were recorded from 24 August 2016 to 25 March 2017. The data were downloaded from the web site under several time units: (i) “PR5m” for periods of 5 min, to compare with VR5m; (ii) “PR2” for periods of 2 h (6 periods per day; 6 periods per night), to compare to VR5m and correlate to IR2; and (iii) “PR12n” and “PR12d”, for periods of 12 h, respectively, for nocturnal records from 18:00 to 06:00, and diurnal records from 06:00 to 18:00, to evaluate the relative impact of diurnal versus crepuscular and nocturnal insects; and (iv) “PR24” for 24 h records.

### 2.5. Statistical Analyses

All data were treated using R software under R-studio environment [37]. Means are indicated with 95% confidence intervals. Mean comparisons were implemented using *t*-test. Insect records (IR) were established as the total of insects trapped by the 3 traps under two time units: (i) IR2: 2 h periods (6 periods a day, or 6 periods a night): IR2 was used to study correlation with PR and VR; and (ii) IR12d: 12 h period day time (from 06:00 to 18:00) or PR12n for night-time (from 18:00 to 06:00) for the analysis of the daily impact of diurnal (and occasionally nocturnal insects). Statistical analysis was conducted using the linear regression model, and interpretation was based on Pearson’s linear correlation (r), *p*-value from the ANOVA and the coefficient of determination of the regression (R^2^). The normality of data distribution was checked using histograms for insect trapping and Q–Q plot for pedometer records. Mean monthly day and night PRs were compared regarding the experiment in order to show the relative impact of nocturnal versus diurnal Diptera.

## 3. Results

**Figure 1:**
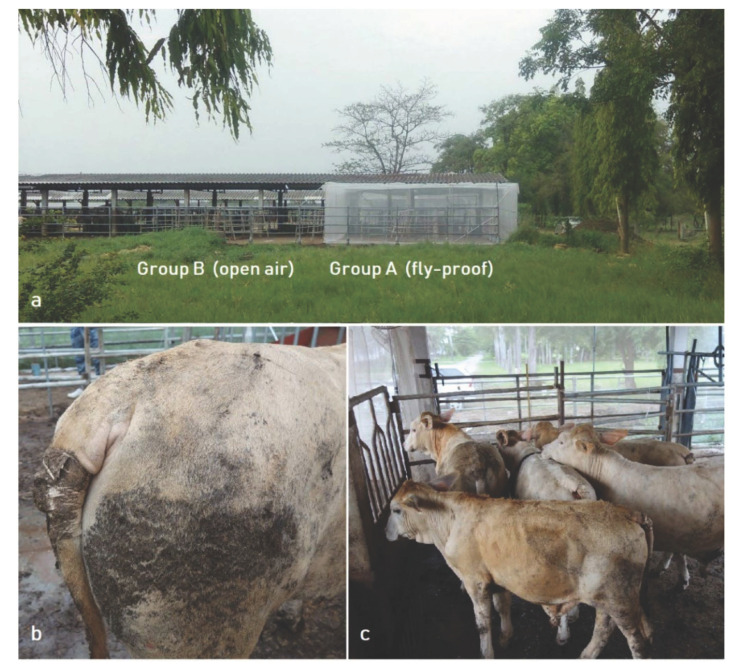
Experimental device of the two cattle groups ((**a**) general view) and pedometers installed on the tail of cattle ((**b**) detail; (**c**) group).

The fly-proof pen was built as shown on Figure 1a, side to side with the open-air pen.

### 3.1. Animals and Grouping

A number of animals were rejected as described under material and method. All the animals proved to be negative to all hemoparasite-detection tests, and remained negative all throughout the experiments. Twelve calves, 9–11 months old, were selected. The mean age of the animals was 299 ± 13 days; there was no significant difference between the two groups (Table 1). The mean weight of the animals was 223 ± 17 kg; there was no significant difference between the two groups (Table 1). Pedometers were attached to the tail of all cattle, as described in the Material and Methods section (Figure 1b,c) and regularly checked, since they were sometime dysfunctional, probably due to bumps, crushing and occasional removals by the animals.

### 3.2. Daily Insect Records

**Figure 2:**
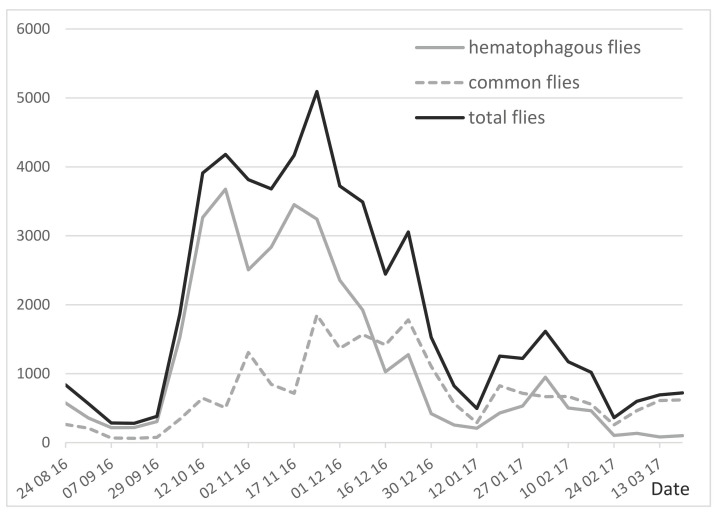
Number of hematophagous and non hematophagous Diptera flies trapped using 2 Vavoua and 1 Nzi traps in one day (IR12), once a week, in the study area (24 August 2016–17 March 2017). **Legend:** Black continuous line: total flies; grey continuous line: hematophagous flies; grey broken line: common flies.

Throughout the experiment, using three traps, the one-day weekly trapping collected 32,770 hematophagous flies (62%), versus 38% common flies (non-hematophagous flies of the sub-family Muscinae), from a total of 53,275 flies (Table 2). Daily insect records (IR12), the total number of insects trapped in a day by the three traps (12 h from 06:00 to 18:00), are a source of basic information which can be used to evaluate the insect burden. As shown in Figure 2, the IR12 (established once a week) was quite low at the beginning of the experiment, until the end of September 2016 (<400 insects/3 traps/day, meaning an apparent density per trap ADT < 130). From that time, the number of insects increased to reach a peak in October–November (ADT > 1300–1700), a period during which insects seriously impacted the behavior of the cattle. Insect density then decreased (ADT < 200) from December 2016 to the end of the experiment in March 2017, although a small peak (ADT = 500) was observed in early February.

Common flies may be annoying for cattle when present in huge density. However, considering that their relative density is lower than that of hematophagous flies, and their annoyance is also lower, the nuisance of non-biting flies may be limited. In this study, we considered the hematophagous flies alone (32,770 specimen caught), due to their specific impact, strong annoyance and high density, since they represented two thirds of the insects caught, including *Stomoxys*, tabanids and *M. crassirostris* (Table 2). The latter is not, sensu stricto, a biting fly, but it is an obligatory hematophagous insect able to draw cattle blood when scratching from the biting site of real biting flies, or a thin fragilized skin area [2]; it was thus considered as part of the hematophagous flies.

Hematophagous flies were predominant with a mean rate of 62%; their weekly percentages ranged from 60–88% in August–November, which decreased to 12–34% in January–March.

### 3.3. Daily Pedometer Records

**Figure 3:**
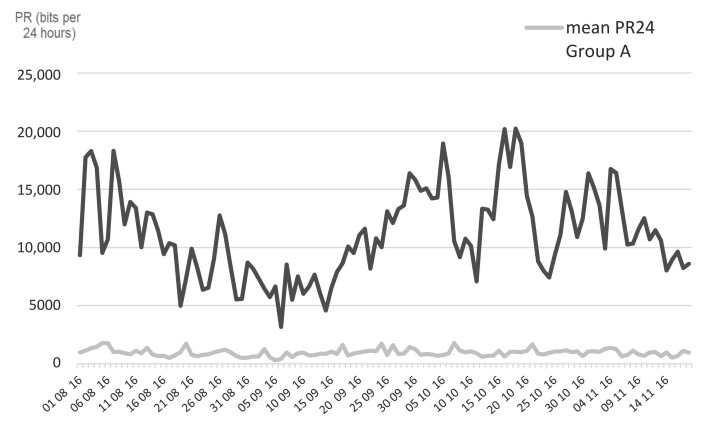
Mean pedometer records per 24 h (PR24) in 2 groups of feeder cattle kept in open-air (Group B) or under fly-proof protection (group A) from August to November 2016. **Legend**: Black line: mean pedometer records (PR24) of cattle in Group B; Grey line: mean pedometer records (PR24) of cattle in Group A.

The pedometers were recording continuously; the mean number of bits recorded per period of 24 h (PR24), along the experiment in Group A and B from 1 August 2016 to 18 November 2016, is presented in Figure 3. Throughout this period, the mean PR24 of Group B (11,138 ± 705 bits) was significantly higher (*t*-test, *p* < 0.0001) than that of Group A (957 ± 58 bots), which shows the high amount of tail flick activity generated by insect annoyance.

### 3.4. Temporal Distribution of Insects

**Figure 4:**
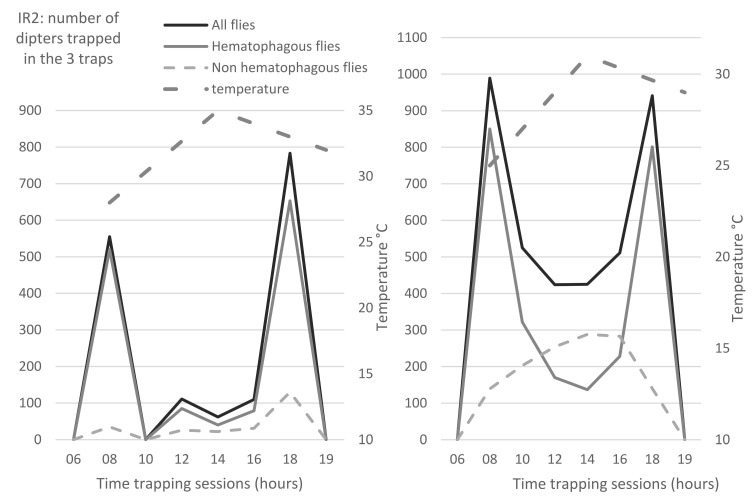
Examples of bimodal daily temporal distribution of flies, from 06:00 to 18:00 and mean temperatures (at 8:00, 14:00 and 19:00) on 17 August 2016 (**a**) and 2 November 2016 (**b**). **Legend**: Black continuous line: total flies; grey continuous line: hematophagous flies; grey broken line: common flies; dot and dash grey line: mean temperature. Arbitrary value of zero was given to the IR at 06:00 and 19:00 to complete the daily graphs. For mean temperatures, data were obtained from the meteorological station of Nakhon Pathom Agromet, Thailand.

The temporal distribution of flies was studied along the day, as well as the variations along the season. During all our observations, biting flies had predominantly a bimodal activity. Hematophagous flies exhibited a peak of activity in the morning and another one at the end of the afternoon, as shown in Figure 4a,b (black broken lines). However, depending on the temperature, relative humidity and rainfalls, other patterns could sometimes be observed (data not shown). Common flies generally exhibited more monotonous activity along the entire day, as shown on Figure 4b (light grey broken line).

### 3.5. Tail Flick Visual Records (VR) and Pedometer Records (PR)

The visual records (VRs) of tail flicks were established between 29 September 2016 and 17 March 2017, once a week, for a total of 21 days of recording. Visual records of tail flicks over 5 min, VR5m, were conducted six times a day, once each 2 h session (from 6:00 to 18:00), which led to a total of 630 min of records per animal (10.5 h of observation) and a total of 7560 min of observation for all 12 cattle (126 h of observation in total). Pedometer records for 5 min (PR5m) were registered between 29 September 2016 and 17 March 2017, once a week, once per time session, for a total of 21 days of recording, at the same time as visual records.

**Figure 5:**
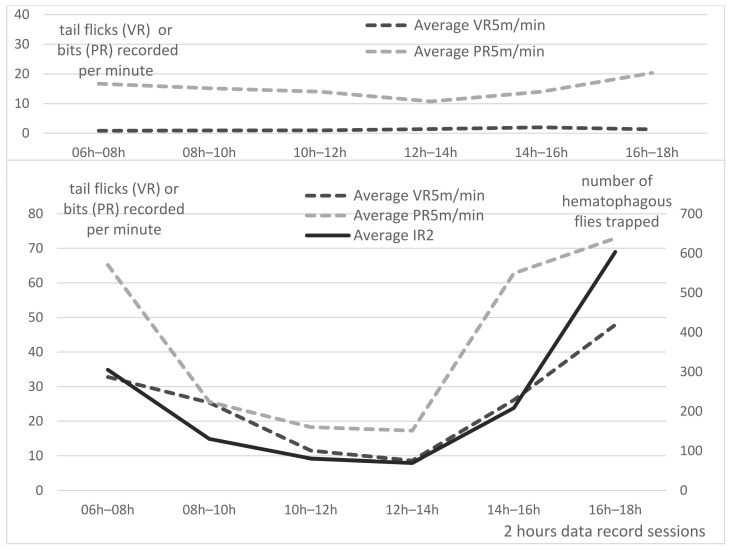
Averaged per minute tail flicks established by visual records (VR5m/5) and pedometer records (PR5m/5) on 6 feeder cattle and insect records (IR2), per 2 h data record sessions, registered for 21 days, from 2 August 2016 to 17 November 2016, in Group A (**up**) and B (**down**).

Averaged PR5m and VR5m per minute of Group A and B are presented per 2 h sessions in Figure 5, with insect density (IR2) as an explanatory factor for group B.

In Group A, visual records of tail flicks (VR5m/5) were very low all along the day: on average, 1.2 +/− 0.8 tail flick/min; however, tail pedometers (PR5m) recorded more “steps” than VR5m, probably due to the animal movements, with a mean of 15.1 +/− 6.3 steps/min. Along the day, higher values were observed in the morning and evening, and are obviously linked with the food-delivery time (07:00 and 17.00).

In Group B, the average visual records of tail flicks per minute (VR5m/5) was much higher: on average, 25.4 +/− 28.2 tail flicks/minutes. Tail flick frequency is very high in the morning and evening sessions, reaching more than 32 and 48 tail flicks/min, respectively, obviously linked with an important fly activity as typically represented on Figure 4a,b. Lower values are recorded at noon (around 10 tail flicks/min). The mean PR5m/5 in Group B was 43.7 +/− 50.8 bits/minute. Similar to Group A, PR5m in Group B exhibited higher numbers of “steps” than VR5m, due to the animal movements and possibly a higher “sensitivity” of pedometers records, since strong tail flicks may induce two to three step records (bits) recorded by the pedometers, while a visual observation of the animal movement would only count for one. Statistical analysis of the correlation between VR5m and PR5m through the Pearson’s correlation coefficient (r) demonstrated a strong correlation (r = 0.873 and *p*-value: 0.023) between the two types of measures in Group B. This strong correlation validates the use of tail pedometer to estimate the tail flick frequency of feeder cattle kept in a pen.

### 3.6. Comparison of 5 min Visual (VR5m) and Pedometer Records (PR5m) Versus 2 h Insect Records (IR2) in Group B

IR2, the total numbers of insects trapped in the three traps during each of the six 2 h trapping sessions in the 21 days of records (once a week from 29 September 2016 to 17 March 2017), are represented in Figure 5 (continuous black line), with VR5m (discontinuous light grey lines) and PR5m (discontinuous grey lines) of Group B. Although the insect’s activity may not always present the same bimodal time-distribution all over these 6 months study, it was mainly bimodal (morning and evening peaks) all along the study period; thus, data collected over 21 weeks were averaged to build this figure.

Statistical analysis of the correlation between VR5m and IR2 through the Pearson’s correlation coefficient (r) demonstrated a very strong correlation (r = 0.944 and *p*-value = 0.010). Statistical analysis of the correlation between PR5m and IR2 through the Pearson’s correlation coefficient (r) demonstrated a strong correlation (r = 0.841 and *p*-value = 0.057). Overall, VR5m exhibited a higher correlation with the insect activity (IR2) than the PR5m.

### 3.7. Comparison of 5 min Visual Records (VR5m) and 2 Hours Pedometer Records (PR2) Versus 2 h Insect Records (IR2) in Group B

**Figure 6:**
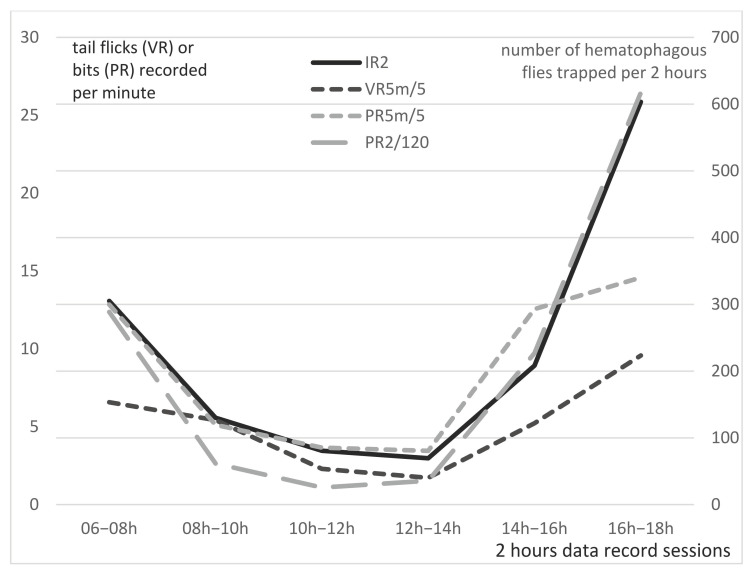
Averaged per minute tail flicks by visual records (VR5m) and pedometer records per 5 min (PR5m) or per 2 h (PR2) in 6 feeder cattle and number of hematophagous flies trapped (IR2), registered for 21 days, from 2 August 2016 to 17 November 2016, for the 6 daily 2 h data record sessions, in Group B.

With the pedometers continuously recording, the total number of “steps” recorded during the 2 h trapping sessions (PR2) was available to further analyze the correlation with IR2. Indeed, a better correlation between PR2 and IR2 was obtained compared to the PR5m (and VR5m) previously analyzed. Statistical analysis of the correlation between PR2 and IR2 through the Pearson’s correlation coefficient (r) demonstrated a very strong correlation (r = 0.993 and *p*-value < 0.0001), as shown on Figure 6. The correlation with IR2 (black continuous line) is stronger for PR2 (grey long broken line) than VR5m (dark grey short broken line) or PR5m (light grey short broken line), which clearly points to the idea that PR generate a better image of insect annoyance than visual counts, especially for high fly density (>300 flies). In addition, the correlation with IR2 is obviously stronger for PR2 than PR5m, which clearly points to the idea that the continuous records provided by the pedometers during the 2 h period generates a better image of insect annoyance than the 5 min samplings generated in PR5m, especially for high fly density (>300 flies).

The strong correlation between VRs and PRs, on one hand, and IRs on the other, demonstrates that pedometers are a good indicator of insect activity, even if the number of bits may overestimate the number of tail flick movements, as was demonstrated in Group A, probably due to other movements of the animals. Continuous records operated by PR2 exhibited a very high correlation with IR2. This strong correlation validates the use of tail pedometer to estimate the biting insect density of feeder cattle kept in a pen.

In addition to the automation of the data, pedometers have the advantage over visual counting (which is time-consuming and provides only discontinuous and sporadic data on cattle defense movements) of being able to record movements continuously, for 24 h a day. This generated a more robust set of information during the day, and interesting additional information at night (see “day and night observations”, hereinafter).

### 3.8. Modeling Pedometer Records (PR) and Insect Apparent Density per Trap (ADT = IR/3)

**Figure 7:**
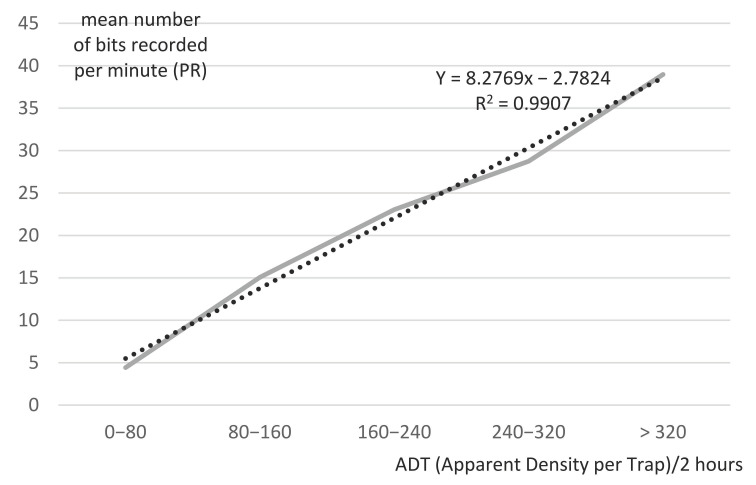
Pedometer record frequencies (bits/minute) in function of the ADT/2 h of hematophagous flies.

Pedometers proved to be highly reliable tools in evaluating insect annoyance, due to their strong link with insect density (IR), obtained from the three traps catching flies in the surrounding area. Figure 7 shows the simple linear regression of the bit frequency recorded by the tail pedometers (PR/min) in the function of the mean apparent density per trap of hematophagous flies, in 2 h trapping sessions (ADT/2 h). The data were obtained from the mean pedometer records (PR2/120) of the animals from Group B, during the six 2 h periods of a day, and the insect records from the three traps divided by 3 (IR2/3 = ADT). The data of ADT were grouped per 80 flies and re-ordered according to a growing ADT/2 h. The data were proven to be in normal distribution based on histogram (for ADT) and Q–Q plots (for PR). The results clearly show an increase of tail movement frequency (PR/min), which is highly linked with the ADT of hematophagous flies. Using a simple linear regression model (y = 8.2769x − 2.7824; R² = 0.9907), 99% of PR/min was determined by ADT/2 h. Tail pedometers can, therefore, be used as a predictive tool to evaluate insect density (ADT).

### 3.9. Day and Night Observations

**Figure 8:**
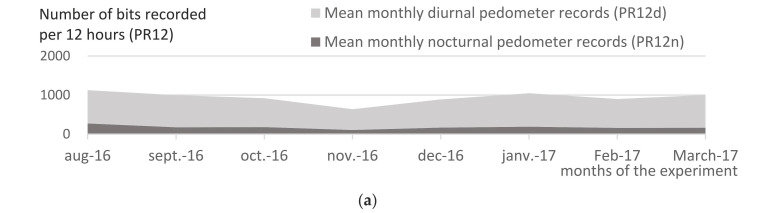

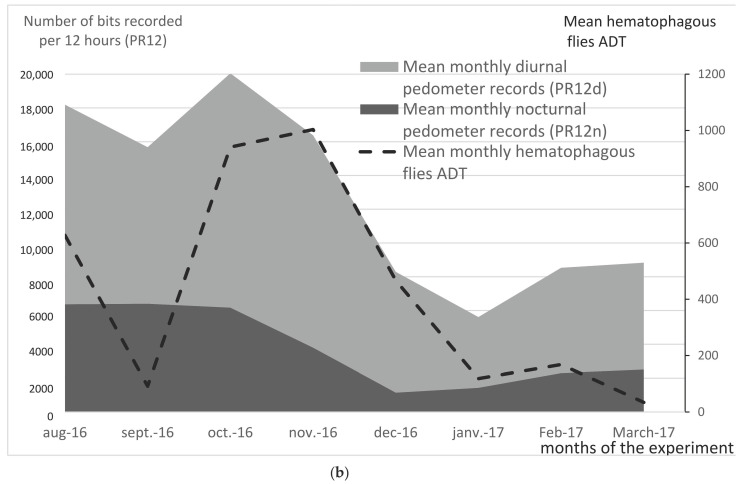
(**a**) Mean monthly nocturnal (PR12n) and diurnal tail pedometer records (PR12d) of Group A. **Comment:** PR12n are constantly below 200/night, while PR12d are constantly around 800/day; there are very light variations along the seasons. (**b**) Mean monthly nocturnal (PR12n) and diurnal tail pedometer records (PR12d) of Group B and mean monthly daily hematophagous fly apparent density per trap (ADT). **Comment:** PR12n (dark grey) are very high (around 6000 bits/night) during rainy season, in August–October and they further decrease down to 1000–2000 bits/night (December–March)t; PR12d (light grey) obviously follow the variations of fly density (black broken line), with high scores from August to November (10,000–14,000 bits/day) and they further decrease when fly density is low in January–March, with scores of 4000–6000 bits/day.

Diurnal pedometer records (PR12d, number of bits recorded from 06:00 to 18:00) and nocturnal pedometer records (PR12n, from 18:00 to 06:00) from Group A and Group B were compared, as well as that of Group B through the seasons. Figure 8 shows the averaged monthly diurnal PR (PR12d, light grey color) and nocturnal PR (PR12n, dark grey color) of Group A (Figure 8a) and B (Figure 8b), from August 2016 to March 2017. Night is a resting period for cattle, as shown by the result of PR12n < 200 bits/ night, compared with PR12d around 800 in Group A (Figure 8a). In Group B, in the daytime, PR12d was 5 to 15 times higher than Group A, ranging from a minimum of 4000 bits/day in low biting fly density season (January), up to 10,000–14,000 bits/day during high-density seasons (August−November). In the night-time, conversely to Group A, PR12n demonstrated an important night-time activity of cattle exposed to Diptera (Figure 8b), with PR12n ranging from 1000 bits/night during the cold, dry season, up to 6000 bits/night during the hot, rainy season. In other words, bit frequencies were 5 to 30 times higher in open-air than under flyproof conditions. The records from Group B showed that the cattle performed a considerable number of tail flicks at night, which could reveal the annoyance of nocturnal insects. This underlines the fact that night tail flicks are far from negligible, especially for the period from August to October–November, the rainy season in this area of Thailand, which is also the season of high mosquito densities. A special nocturnal insect trapping session was then conducted in November 2016; trapping scores are reported in Section 3.10.

### 3.10. Night Insect Trapping

**Figure 9:**
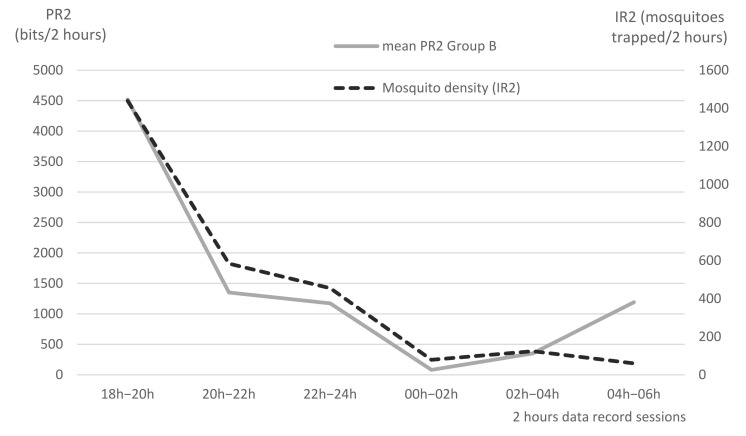
Temporal distribution of mosquitoes trapped (IR2) and pedometer records (PR2) in Group B, for one night (21–22 November 2016) per 2 h data record session.

Using the CDC trap hung upon Pen B, the trapping box was changed every 2 h from 18:00 on 21 November 2016, to 06:00 on 22 November 2016. Mosquitoes were the most representative nocturnal insects (88%) over this night, with a total of 2741 specimens trapped. An obvious relation can be seen between mosquito density (IR2) and tail flicks per 2 h session (PR2) in the last two columns of Table 3, and on Figure 9. It can thus be concluded that mosquitoes were responsible for the high PR2 observed at early night. Very low PR2 values were observed in the middle of the night, when mosquito trapping records were also very low. The increasing PR2 value in the early morning (1192 bits between 04:00 and 06:00) shows some discrepancy with the recorded mosquito density, which was low. However, the increasing PR in the early morning may be due to the activity of biting flies (*Stomoxys*), rather than mosquitoes, even if biting flies were not trapped by the CDC. Indeed, the CDC shows that it had a very low attractivity in the early morning, due to the interference of the natural light of sunrise.

Overall, the effect of mosquito bites may have a significant impact on cattle welfare and production.

## 4. Discussion/Conclusions

Tail flicks were reported by several authors to be a reliable behavioral response of livestock to biting flies [17,19,20]. This study aimed to evaluate the efficiency of tail pedometers for the automation of tail flick counts in two groups of six feeder cattle kept in pens (14 sqm/head), either in open-air conditions (Group B) or under the protection of a mosquito net (Group A).

Pedometers were not always easy to maintain on the tails of the cattle and were regularly checked and replaced, due to being broken or lost. Thirty-two pedometers were used for six animals over a 7-month period (five to six per head, on average), which corresponds to a total cost of around 3000 USD. Using pedometers allows us to generate a huge quantity of reliable data with reduced working time. Generating the same quantity of data by visual records would require around three full time (3 × 8 h/24 h) technicians per head (3 × 12 = 36 persons) for the duration of the study (7 months) and the cost of this would be prohibitive.

However, pedometer records may not always be as reliable as in the present study. Indeed, in further unpublished experiments, when pedometer records were made using free or attached milking cows, the correlation between biting fly density in the surrounding area (IR) and pedometer records (PR) was sometimes much lower. This observation suggests that breeding conditions and the physiological status of the animals may severely impact cattle behavior and, consequently, severely affect the reliability of tail flick records as a tool to estimate insect density and impact. A careful selection of cattle, as was conducted in this study, is necessary to avoid non-representative behaviors (linked to hyper or hypo-reactivity/passivity, aggressivity, excitation, estrus, etc.).

The mean numbers of tail flicks/minute reported or estimated in this study (averaging from 5 to 45) were quite compatible with reports previously published by a number of authors [17,38,39,40]. Dougherty et al. (1993) reported around 36 tail flicks/minute, and they showed a linear relation between the number of biting flies released in a screened enclosure and the number of tail flicks of grazing beef cows. In the present study, we also demonstrated a very strong correlation between fly density and tail flick frequency. Mullens et al. (2006) produced similar results, and they reported that tail flicks were not effective to repel the flies but were well suited to quantify fly density. El-Laithy (2007) observed that tail flicks, averaging 15/min, were the main fly avoidance behavior exhibited, followed by ear flicks (11.5/min) and skin twitches (10/min). In a subsequent study on buffaloes, El-Laithy (2013) recorded around 19 tail flicks/min, followed in frequency by ear flicks (16.5/min).

This study is the first to report an estimation of the total number of tail flicks per 24 h in exposed or non-exposed cattle, ranging from 11 thousand bits per 24 h in open-air conditions to below one thousand under fly-proof conditions. The scores of pedometer records (PRs) related to the. basic activity of the cattle were below 200 per night and 800 per day under fly-proof conditions, while these ranged from 1000–6000 at night and 4000–14,000 per day in cattle exposed to open-air conditions. Definitively, PR were excellent indicators of Dipteran activity, during both the night- and day-time (see below for night-time).

On a daily basis, biting flies mostly exhibited a bimodal activity, as demonstrated by the 2 h insect trapping sessions. This is in accordance with the observations made by Raymond in French Guyana [20], and more generally under hot weather conditions [41,42,43], when the temperature at noon is over 30 °C. These results are different from those of El-Laithy 2013 [40], who reported maximum activity at noon and in the afternoon; however, this was due to the slow rise of the temperature in their study area.

The number of insects trapped per 2 h session (IR2) was strongly correlated with VR5m (5 min visual records) and PR2 (2 h pedometer records); the correlation of pedometer records with insect activity was the strongest (r = 0.99 for PR2/IR2 versus r = 0.94 for VR5m/IR2). These results validate the use of pedometers both for tail flicks and insect density estimations. Based on the data collected, a strong linear relation was drawn between the apparent density per trap (ADT) of hematophagous flies, and the number of bits recorded per minute (PR/min), which allows a prediction of the ADT around cattle to be made with an accuracy of 99%.

Thanks to the continuous recording enabled by pedometers, an unexpected activity was reported at night. The night trapping, implemented using a CDC trap, allowed us to establish a strong correlation between mosquito density and pedometer records.

When considering the night and day additional bits induced by insects (Group B versus Group A), throughout the experiment, 62% of the defense movements could be attributed to biting flies, and 38% to mosquitoes (percentages extracted from the data presented in Figure 8b). This observation draws interest to the nuisance of mosquitoes in cattle, the impact of which, neglected in research thus far, should be more thoroughly investigated. Indeed, most of authors used to consider mosquitoes to not be important pest for livestock, as stated by, for example, Hill: “The feeding adults will take some blood and cause some irritation, but not usually a great deal” [44]. Nocturnal insects’ population and behavior are not well-known in cattle. Furthermore, in dairy cattle farming systems around Nong Pho, Thailand, all farmers agree that nocturnal insects are a serious pest for cattle at some period of the year. Further studies are therefore suggested to determine the impact of mosquitoes in cattle and possibly other livestock. Mosquito control in livestock might also be justified.

## Figures and Tables

**Table 1:** Mean age and weight of the animals of Group A and B and *p*-values of *t*-test.

	Group A	Group B	*p*-Value
Mean body weight ± 95% CI (kg)	222 ± 29	224 ± 20	0.93
Mean age ± 95% CI (days)	300 ± 18	299 ± 21	0.94

**Table 2:** Diurnal dipteran flies trapped during the study 24 August 2016–24 February 2017 (trapping sessions from 06:00 to 18:00) in the three traps (2 Vavoua and 1 Nzi).

Flies Trapped	Number of Flies Trapped	Percentage
Tabanids	837	2%
*Stomoxys* spp.	27,839	52%
*Musca crassirostris*	4094	8%
Total hematophagous flies	32,770	62%
Common flies (other *Musca* spp.)	20,358	38%
Total flies trapped	53,128	100%

**Table 3:** Insects trapped with CDC trap at night per 2 h sessions on 21–22 November 2016 and mean PR2 of cattle in Group B.

Time	Total Insects Trapped/2 h	*Stomoxys*	Common Flies	Other Non-Biting Insects	Mosquitoes	Mean PR2 Group B
6–8 p.m.	1557	7	5	106	1439	4520
8–10 p.m.	702	9	12	97	584	1351
10–12 p.m.	560	0	7	98	455	1172
0–2 a.m.	90	0	0	11	79	81
2–4 a.m.	131	0	0	7	124	352
4–6 a.m.	80	0	0	20	60	1192
total	3120	16	24	339	2741	8668

## Data Availability

Data available on request from the corresponding author.
